# Bridge Over Troubled Water: Perspective Connections between Coping and Play in Children

**DOI:** 10.3389/fpsyg.2016.01953

**Published:** 2016-12-26

**Authors:** Michele Capurso, Benedetta Ragni

**Affiliations:** ^1^Department of Philosophy, Social Sciences and Education, University of PerugiaPerugia, Italy; ^2^Associazione Gioco e Studio in OspedaleGenova, Italy

**Keywords:** children, play, coping, appraisal, multidimensional, emotional regulation

## Abstract

We propose that children’s play and coping strategies are connected. However, this connection has often been overlooked in the literature. To prove our hypothesis, the principal developmental functions of play are reviewed and compared with the different stages of the coping process. Our results show that coping and play are essential elements in child development, and indicate the presence of several overlapping areas where play and coping intersect. In spite of this, their interrelationship has seldom been examined. We explore the possible reasons for this omission with reference to the different natures of play and coping constructs, and also to the definitive psychometric and cognitive characteristics of most common coping measurement instruments. We conclude by proposing that play should be considered an elective form of coping in most aspects of children’s lives. We also propose that methods to measure coping in children should be improved and a more analogical approach should be adopted toward play to enable accurate recognition of coping.

## Introduction

Coping and play are widely recognized as being crucial to child development. In different ways, they both support mental, physical, social, and emotional well-being and the ability to adapt (Bjorklund and Pellegrini, 2002; Russ, 2004; Ginsburg, 2007; Skinner and Zimmer-Gembeck, 2007; Aldwin, 2009; Zimmer-Gembeck and Skinner, 2016). Here, we aim to highlight limits in current coping theories that obscure the importance of play and show possible future developments for integrating both constructs.

## Coping

Coping is a strategy employed to manage and adapt to stressful and ever-changing environments and situations (Lazarus and Folkman, 1984). Stressors and coping responses vary throughout life and appear to be linked to the individual’s appraisal of the situation, the type of problem faced, socio-cultural aspects and the developmental stage of the subjects (Losoya et al., 1998; Skinner and Zimmer-Gembeck, 2007; Clauss-Ehlers, 2008; Aldwin, 2009).

According to a developmental perspective, coping is a regulatory processes mediating the interrelation between the individual and the environment (Compas et al., 2001; Zimmer-Gembeck and Skinner, 2016), and it characterizes how children face and respond to stressors both in adaptive and maladaptive ways (Zimmer-Gembeck and Skinner, 2016). The adaptive nature of coping is fundamental for human wellbeing; as outlined by Lazarus and Launier (1978), a coping response is even more important than the stressor itself. Effective coping has been associated with important outcomes in childhood and adolescence, such as academic performance, social functioning, adaptation to stressful life events, internalizing and externalizing behavior, well-being, competence, and resilience (Zimmer-Gembeck and Skinner, 2011, 2016).

### Normative Development of Coping

Coping adapts and develops as a joint function of internal traits and environmental characteristics (Skinner and Zimmer-Gembeck, 2007; Zimmer-Gembeck and Skinner, 2016). Infants engage in reflex actions mediated by their own temperament (Rueda and Rothbart, 2009), and volitional coping strategies start to emerge from early childhood (Compas et al., 2001). As children mature, their coping strategies develop as follows:

• Initial stage of social referencing (Klinnert et al., 1983; Fonagy et al., 2007), where children quickly attune to their caregiver’s reactions to assess potential dangers and whether they should engage or withdraw from the external situations;
• Concurrent stage of interpersonal coping, in which children intentionally instigate coping actions in their caregivers through by communication aimed at producing the desired results (Zimmer-Gembeck and Skinner, 2016);
• Predominance of distraction strategies in younger children and progressive differentiation of this trend in children over 4 years old (Zimmer-Gembeck and Skinner, 2011).
• Gradual shift from behavioral actions to more cognitive-based and emotion-focused forms of coping (Losoya et al., 1998; Spirito, 2003);
• Increase in problem-solving and the ability to regulate the coping response according to the stressful situation (Zimmer-Gembeck and Skinner, 2011);
• Development of regulation strategies that in turn increase the use of emotion-focused forms of coping from age 6 onward (Band and Weisz, 1988; Altshuler and Ruble, 1989; Aldwin, 2009);
• Increased seeking for social support and the shift from parent-centered help to peer support, especially for emotional problems (Crystal et al., 2008).

### Developmental Limits of Current Coping Models

The main limitation of current studies into children’s coping strategies is the tendency for children’s strategies be regarded in the same way as those of adults. This is caused by three main factors.

The first is the persistence of many analytical tools that are either directly or indirectly derived from adult-driven instruments and pre-existing theories (Ryan-Wenger, 1992; Moreland and Dumas, 2008). As a result, coping categories for children are not based on directly observed realities, but rather on the views of other scholars. Such theory-driven categories overlook the real situation and prevent researchers identifying aspects of behavior that do not fit with what is expected (Ford and Lerner, 1992; Telfener, 2011; Monette et al., 2013). The limits of this approach were shown in an interview-based research conducted by Band and Weisz (1988), where approximately 40% of the children’s responses fell outside of the coping categories used in adult studies, and new specific categories had to be established to properly classify the children’s responses. Compas et al. (2001, p. 87) argued, “The way in which coping is conceptualized influences methods of measurement and defines the scope of what is included within the rubric of coping. Many of the problems in the field have come from the lack of clarity and consensus regarding the nature of coping during childhood and adolescence.”

This leads to the second factor. If the construct of coping is cognitive in nature, then children’s coping tools should align with their cognitive developmental stage, as defined by current theories in the field. For instance, if the coping strategy of a group of children in the preoperational stage, as defined by the Piagetian theory, is to be measured, then the tool should consider egocentrism, symbolic play, animism, irreversibility, etc.

Finally, many of the coping categories identified by the current measurement tools are mutually exclusive (Ryan-Wenger, 1992; Skinner et al., 2003), where a specific action is interpreted only as a single type of coping strategy (e.g., “go out and play” is only classified as distancing from the stressful event). While such an approach serves the need of a taxonomy-based construct (Reynolds, 1971), it inevitably fails when faced with the multidimensional characteristics of play, leading to a partial and incomplete consideration of its potential.

### Play: A Neglected Aspect in Coping Studies

As a consequence of the above-mentioned limits, the role of play has often been neglected in coping research. A review by Capurso and Pazzagli (2015) showed that out of 40 studies on coping in children, play was either disregarded or only considered as an avoidant or distracting activity. Additionally, a search for “play” in some of the most recent reviews and theoretical papers on coping and child development (i.e., Ryan-Wenger, 1992, 1996; Fields and Prinz, 1997; Losoya et al., 1998; Compas et al., 2001; Zimmer-Gembeck and Skinner, 2011, 2016), has not revealed any meaningful discussion of the term. Play is often not mentioned, or when it is, is only seen as an attempt by the child to distance themselves from the stressor or delay the need to face the situation.

## Coping Functions are Present Within Play Theories

Conversely, the adaptive and evolutionary functions of play have been studied for many years. Ellis (1998) asserted that play is a biological function to mediate adaptation to unpredictable threats, whilst others suggest that play is a crucial component in children, representing central evolutionary and natural values that continue throughout life (Sutton-Smith, 1997), and mediating adjustment during childhood (Bjorklund and Green, 1992). Given the characteristics of these two constructs, the three main areas where play and coping encounter are cognitive, social and emotional.

### Play as a Means of Cognitive Adaptation to Reality

The benefits of play for cognitive development have long been recognized (Bergen, 1998). Piaget (1951) suggested that play could consolidate skills and thus engender confidence and a sense of mastery. In younger children, play enables them to assimilate everyday experiences into existing schema, act out established behaviors, and adapt reality to their own thoughts. Exploratory play is the basis for learning, achieving goals, and growth (Jambor and van Gils, 2007; Fromberg and Bergen, 2015). As children develop, play increases in complexity, reflecting the maturation of the brain and its functions (Gordon, 2014). Bruner (1983) defined play as an activity without frustrations in which children explore and organize the world according to their desires and experience pleasure in overcoming obstacles. These aspect of play are connected to the appraisal stage of coping, where children assess the external reality to identify and later trial solutions in a way that is at the same time safe and not frustrating.

### Play as a Mediator with a Social and Cultural World

Vygotsky (1967) examined how play promotes self-regulation and learning of cultural values. He considered play as a way to for children to realize their wishes in terms of their cognitive development because it facilitates symbolic representation of the wider socio-cultural world. Following Vygotsky, others observed that children learn how to set limits in a play setting, using symbolic thinking, planning, role-taking, and self-regulation (Bergen, 2015). This aspect also connects to the contextual nature of coping adaptation, where the efficient adaptive responses to stress need to take proper account of the local culture and social context. In addition, play has also been linked to more formal cultural expressions such as academic learning. Roskos and Christie (2001) outlined the role of play in the development of literacy, while Cook (2000) saw that preschoolers could be effectively engaged in playful activities related to mathematical concepts.

### Play and the Emotional Processing of Stress: The Transitional Space

Scholars have previously outlined the importance of play as a mean of connecting with our emotions. Sigmund Freud (1960) saw play as a way for children to realize their wishes and to overcome traumatic events, providing a safe context to express impulses that are too dangerous to vent in reality. A similar view was shared by Erikson (1993) who considered play as a way to deal with emotional and behavioral dilemmas; Anna Freud (1928) studied how children’s play fosters the ability to face trauma such as war or parental separation.

According to a psychodynamic view, the deep nature of play is ambiguous. Winnicott (1971) explains this by placing play in a special transitional area, where fantasy and external reality coexist. Such a space represents the transition from omnipotence, where the child feels that they can create the world, to an objective and sometimes frustrating reality, where the world is beyond their personal control. Winnicott (1971, p. 51) states that “into this play area the child gathers objects or phenomena from external reality and uses these in the service of some sample derived from inner or personal reality.” The transitional space is in the middle ground between the child and the environment, where the mind moves free and is creative, exploring different possible scenarios connecting fantasy and reality.

### The Coevolutionary Multiplex of Play Functions Meet the Contextual Nature of Coping

In general theoretical terms, the development of play theory corroborates the ambiguous and multifaceted nature of play. After considering hundreds of children’s games in New Zealand (Sutton-Smith, 1959, 1975) and analysis of more than 135 play concepts (Sutton-Smith, 1997), the developmental psychologist and play theorist Sutton-Smith concluded that play was a “Coevolutionary Multiplex of Functions” (Sutton-Smith, 2008, p. 111), where play was recognized as having multiple characteristics across a variety of relevant functional domains that are “genetic, affective, performative, experiential, and culturally relative” (Sutton-Smith, 2008, p 116). While this later view may render a definition of play relative and therefore hard to attain universally, it has the advantage of allowing for a wide set of different play settings, aims and function over time. Such characteristics make matching with the contextual nature of coping constructs possible, since also coping is linked to individual and socio-contextual traits.

## Empirical Contributions Connecting Play and Coping

Several empirical studies of play show meaningful connections with coping in children. (Russ et al., 2000; Russ, 2004) maintained that the cognitive, affective, and interpersonal processes in play mediate key developmental capabilities such as creativity, problem solving, coping, and prosociality. A psychometric scale to measure children’s ability to process emotions through play was developed and the researchers suggested that in fantasy play children can create controllable events while venting negative emotions. This increases positive feelings and reduces anxiety (Christiano and Russ, 1996; Saunders et al., 1999; Lester and Russell, 2008). Russ (1999, 2004) and Strayhorn (2002) suggested that when children play within a creative framework, they are actually testing adaptive solutions on cognitive, behavioral, and emotional levels, and this practice is linked to their ability to cope with difficult situations in everyday and later life. Rutter (1985) argued that play develops adaptability and flexibility, supporting children in facing stressful situations, which is also one of the main purposes of coping strategies. Tegano et al. (1989) and Schaefer and Drewes (2010) recognized the importance of play as a mediator of coping. They suggested that the lack of fear of any real consequences and the autonomy experienced in play allowed children to develop new solutions, helping them to comprehend and solve their social and personal problems.

Social play is another key aspect connected to coping. This involves a child taking on different roles and increases the development of communicational skills, problem-solving and empathy (Hughes, 2009). Free social play is related to the ability to understand others’ viewpoints, helping, cooperating, sharing and problem-solving (Gleave, 2012). Conversely, children who had lost their capacity for creative play after a trauma were hindered in forming social relationships and showed decreased problem-solving capabilities (Lovett and Boyd Webb, 2010). Play can be associated with coping because once the game is over, the lessons remain: new learning and understanding “endure as a new found creation of the mind, a treasure to be retained by the memory…,” and becoming “… a cultural phenomenon” (Huizinga, 1949).

An emblematic setting where different kinds of play have repeatedly shown coping potential is in a hospital. For example, Haiat et al. (2003) and Hubbuck (2009) showed that play with puppets helps children to understand medical procedures, creating a positive attitude toward the stressful situation. They also recognized the importance of spontaneous play, especially in children’s ward, as this could help the children to understand and consolidate new or complex information. To properly recognize the role of narrative and fantasy in helping children deal with the stressful circumstances of a chronic illness, Clark (1998, 2003) coined the term “imaginal coping.” The most relevant imaginal coping activities identified are fantasy play, role- and role-reversal play, rituals, stories, humor, and prayers (Clark, 2003; Rindstedt, 2014), all of which are usually carried out with some level of social interaction. Ritual play as an active response to anxiety and health care procedures is further analyzed by Gaynard (2016), who recognizes its function as a holder for- and molder of, feelings.

## Connecting Play and Coping: Theoretical Facets

There are several areas where play can be connected to coping. In children at the preoperative and concrete stage of development (ages 3–10), play may act as an initiator of forms of conjectural thinking. To visualize different solutions and assess different coping actions, in situations of distress, adults start a chain of “if-then” mental statements (Lazarus, 1991); whereas children, lacking the ability of abstract thought, can “pretend-play about it,” envisioning solutions and testing them in a safe and imaginative context. Additionally, in pretend play young children show signs of understanding others’ thinking and beliefs when confronting reality, which is a key component commonly found both in coping and in play activity.

**FIGURE 1:**
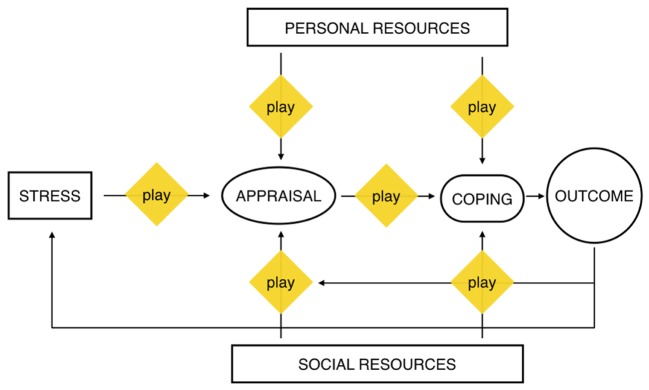
**Coping as a transactional process (adapted from Zimmer-Gembeck and Skinner, 2016) integrated with play functions**. Original Picture Copyright © 2015 John Wiley & Sons, Inc. All rights reserved.

**Figure 1** shows the different stages of the coping process (Zimmer-Gembeck and Skinner, 2016) and how play can act as a mediator at different times. It begins with play mediating the different components in the appraisal phase (the stressor, the assessment of personal and social resources and the potential coping strategy itself) and it then continues with play mediating the coping action with personal and social resources. These aspects should all be the focus of future research.

Because play is a widespread means of communication and a natural language for children (Drewes and Schaefer, 2010; Landreth, 2012), it makes universal communication possible, regardless of their linguistic and cultural differences. Play also offers a natural mediator when working with young people in distress, even if they are from diverse cultures or backgrounds (O’Connor, 2005). Proper consideration of play should not attempt to reduce distinct playful behaviors to a single coping category; rather, to fully appreciate the potential of play as a coping mechanism, its multifaceted and multidimensional nature should be recognized (Huizinga, 1949; Sutton-Smith, 1997). This peculiar characteristic of play should be acknowledged and accepted in any investigation of children’s coping behaviors and strategies.

## Future Research and Open Questions

The relationship between the assessment of children’s coping strategies and play calls for further research in two main areas.

From a theoretical viewpoint, despite studies relating children’s coping to play behaviors (e.g., Russ, 1988; Prinstein et al., 1996), there is a need for a framework to explain the relationship between play and coping. Finding this type of connection is difficult due to the profound disparities in the nature of these two aspects of human behavior. The free and intrinsic origins of play relate to a free and unregulated world; it is ambiguous and its expressions vary with culture, age and location (Winnicott, 1971; Sutton-Smith, 1997, 2008). Coping is related to volitional cognitive processes, and is generally categorized using mutually exclusive taxonomies.

Regarding the psychometric evaluation, future investigation should address the creation of coping instruments capable of operationalizing and validating the role of play and its multifaceted nature. Systematic observations of play sessions during assessments of coping skills would be useful, following the example of several scales developed to measure affective expression and self-regulation in children (Shapiro et al., 1994; Russ et al., 2000). Appropriate categories for play assessment could be connected, in a non-exclusive way, with established coping categories in children, such as those described by Zimmer-Gembeck and Skinner (2011).

Play is a fundamental part of a child’s life, while autonomous coping skills become increasingly important in adulthood. Coping is akin to walking across a bridge over troubled waters, but play shows children different options and enables them to choose which route to take. The metaphor of our title recalls the power of play as a mediator helping the children to work out coping solutions; and the role of play as a mediator between coping and the stressors. The aim of this paper was to connect the free and fantastical world of play with the more rational and grounded theory of coping in children. In doing so we focused mainly on the interconnections of play and coping, outlining the adaptive functions coming from the unregulated characteristics of play. There are, of course, types of play and settings where play is indeed much more structured and this also helps children to cope. In the end, play remains a universal language and a means for children to express themselves. A proper connection with coping will help more children to develop and become more resilient when facing adversity, and this is surely a field worth exploring for any researcher in human development.

## Author Contributions

MC initiated and planned the outline of the article, conducted the research on children’s coping strategies, performed the comparisons of coping and play, and discussed the differences between the two. He also edited the main text of the article. BR researched children’s play and drafted the paragraph about play and child development. She also reviewed the manuscript critically and contributed to the discussion.

## Conflict of Interest Statement

The authors declare that the research was conducted in the absence of any commercial or financial relationships that could be construed as a potential conflict of interest.

The reviewer AL and the handling Editor declared their shared affiliation, and the handling Editor states that the process nevertheless met the standards of a fair and objective review.
